# Chronic Cervicitis

**Published:** 1908-04

**Authors:** T. M. Monroe

**Affiliations:** Center, Mo.


### Chronic Cervicitis.

BY T. M. MONROE, CENTER, MO.

This is a condition which very frequently confronts the general practitioner and gynecologist. Leucorrhea, a symptom which many ignorant persons wrongly consider a disease, is due in a large percentage of cases to a large increase in the amount of cervical discharge. Two kinds of infections attack the glands of Naboth-gonorrheal and simple micro-organism found in the vagina. The first variety occasions a purulent discharge which may appear only at times when the infected gland discharges its contents. The second variety begins, as a rule, when small retention cysts form in the cervix. Retention and decomposition of gland material sets up a low grade inflammation which may persist for years and prove very difficult to cure. Chronic cystic cervicitis is characterized by thick translucent glairy discharge.

In general the treatment of this disease is surgical and local, although in certain cases where the patient's condition is one of lowered vitality, hygienic conditions should be improved and tonics employed.

Free drainage should be afforded the infected structures, and this can best be accomplished by puncturing each distended gland, which feels like a shot or pea to the palpating finger, with the tip of a bistoury, allowing the escape of purulent or glairy material.

Tincture of iodine should be painted over the surface after scarification and douches twice daily, together with applications of mild astringent antiseptics, should effect a speedy cure. In such conditions, Katharmon is very valuable since it is non-irritating, and prevents septic decomposition. Among other ingredients it contains hydrastis, phytolacca, borosalicylic acid and sodium pyroborate dissolved in pure distilled extract of witch hazel. Hydrastis is a valuable alterative, astringent and antiseptic, when applied to diseased mucous membranes, and phytolacca exerts an abative influence on beginning inflammations.

Borosalicylic acid and sodium pyroborate are the efficient antiseptics, disinfectants and deodorants, and witch hazel possesses the tonic and astringent properties possessed by tannin. Thus the value of Katharmon is readily understood when the physiologic effects of its constituents are borne in mind.

